# The rise in HIV cases in Pakistan: Prospective implications and approaches

**DOI:** 10.1016/j.amsu.2022.104492

**Published:** 2022-08-24

**Authors:** Wajeeha Bilal Marfani, Hira Anas Khan, Mahnoor Sadiq, Oumaima Outani

**Affiliations:** Faculty of Medicine, Dow Medical College, Dow University of Health Sciences, Pakistan; Faculty of Medicine and Pharmacy of Rabat, Mohammed 5 University, Morocco

## 1 Commentary

Human Immunodeficiency Virus (HIV) is a retrovirus whose primary target are CD4^+^ immune cells, with the HIV1 strain being the most dominant. At its last stage, HIV causes the Acquired Immunodeficiency Syndrome (AIDS) [1]. HIV/AIDS is transmitted through the exchange of body fluids including semen, blood and breast milk. The main forms of transmission are sexual contact, from the mother to the fetus, breast feeding and sharing syringes [1].

The first reported cases emerged in the 1980s and it has since then affected over 75 million people around the globe, and approximately 3.4 million are estimated to be living with HIV at the end of 2021 [2]. The Global Burden of Disease (GBS) report of 2013 yielded that HIV is one of the leading causes of morbidity and mortality globally [3]. This virus mainly affects the young and economically productive population, therefore has drastic economic and social consequences.

In Pakistan; a large country in South Asia, and as of June 2019, 24,331 people living with HIV (PLHIV) are registered with the National AIDS Control Program (NACP), but the estimated number of people living with the infection is estimated to be 165,000 ^4^. The percentages of vulnerable populations where the HIV epidemic in Pakistan is concentrated are: 38.4% in people who inject drugs (PWID), 7.5% in transgender sex workers (TGSW), 7.1% in transgender people (TG), 5.6% in male sex workers (MSW), 5.4% in men who have sex with men (MSM), 2.2% in female sex workers (FSW). The majority of PLHIV are based in Punjab (75,000) and Sindh (60,000) [5].

Pakistan witnessed several outbreaks in the last few years, namely the Larkana outbreak in 2019 [4]. Larkana is a district in Sindh where officials from the world health organization (WHO) declared a grade 2 emergency; According to the WHO a grade 2 health emergency is a multiple or single country emergency that requires moderate response by the WHO, the response exceeds the capacity of the WHO country office [4]. The drivers the WHO officials identified are mainly iatrogenic such as male circumcision tools, reused needles and unsafe blood transfusions.

The curative and preventive efforts against HIV in Pakistan declined because of the current COVID-19 pandemic [6]. A Lancet global health modeling study found that HIV deaths are expected to rise by 10% in Low- and middle-income countries over 5 years because of the disruptions the pandemic cause [6].

In this paper, we first discuss the different international and national challenges the Pakistan's healthcare system faces and that compounded to cause the current rise in cases, we then discuss the efforts being led by the various stakeholders to tackle the crisis, and finally we highlight recommendations that can be applicable in the local context and that can further assist in addressing this rise.

As HIV is a rapidly mutating virus, the development of a single vaccine to curb all HIV strains serves as a major challenge to human society in recent times [7]. Antiretroviral treatment (ART) is the only available regimen currently. Approximately, 147,851 individuals in Pakistan do not have access to it and 7182 patients missed a follow-up visit in the last 6 months [8,9] usually because of the social stigma and resistance to accepting the existence of non-marital sexual activity, which hinders patients from reporting symptoms at clinics, drawing scorn from families and even physicians. ART availability and adherence are severely hampered by the rising number of new HIV patients and great travel distances to seek medical treatment as there is a lack of expertise to run ART clinics [10]. Due to the rising annual rate of inflation [11], the ARTs are running out of stock as they are not manufactured in Pakistan and must be imported. HIV drug resistance is also a critical global health issue [12] that can affect HIV treatment in countries with fragmented healthcare systems, shortages of second-line ART regimens, and suboptimal virus surveillance.

The extensive use of unsterilized medical instruments by untrained healthcare staff, and local quacks act as a catalyst for the spread of HIV and other blood-borne illnesses including hepatitis B and C [13]. Data from a study by Davlidova et al. reveals that Larkana (located in the Sindh province of Pakistan) police filed a complaint against 24 private practicing physicians involved in cases of medical negligence [14]. Another study reports that in the Kot Imrana village (located in the district of Sargodha in the province of Punjab, Pakistan), 96% of HIV patients documented a lack of understanding of its transmission, it was also recently confirmed that a quack (traditional healer) who died of AIDS in 2018 was using infected syringes and was responsible for contaminating hundreds of people in this village [15]. Similarly, in Sargodha 5000 quacks may be infecting the patients with HIV/AIDS [16].

The current data suggests that in Pakistan there is a dearth of effective systematic surveillance programs and strict policies to halt HIV spread and accurate evidence-based statistics are missing. The country is also lacking in providing adequate budgets, rehab centers for injecting drug users (IDUs), vigilant therapies for the vulnerable population, and contact tracings [17,18]. This lack of multidisciplinary measures, absences of willingness, and ignorance from healthcare sectors can give rise to new variants that might be difficult to combat.

In terms of the HIV epidemic, Pakistan is the second largest country in South Asia after India and Nepal [19]. Recent years have seen a significant increase in HIV infections despite many efforts [19]. HIV prevalence is higher than 5% in at least eight major cities among injecting drug users (IDUs), shifting from a low to a concentrated epidemic [19].

As part of the new HIV prevention program launched in Pakistan in 2018, community-based organizations are working to reach the most at-risk people, including injecting drug users, sex workers, transgenders, and gay men [20]. In addition to HIV testing, mobile testing vans provide counseling, information about HIV and how to prevent it, links to HIV treatment if needed, and condoms, lubricants, and treatments for sexually transmitted diseases [20].

It was in 1987 that Pakistan's Federal Ministry of Health launched its National AIDS Prevention and Control Program (NACP) [21]. NACP currently working in parallel with the National Institute of Health to achieve its goals [19] promotes the registration of all HIV-positive individuals, engagement of high-risk groups via community-based organizations, and free HIV testing and treatment to achieve universal access to antiretroviral treatment (ART) [8]. In the beginning, the program primarily focused on blood screenings and health promotion, and HIV education. However, the strategy failed to address high-risk populations and there was inadequate surveillance and research to inform decision-making [21]. A broad consultation process then led to the development of the National Strategic Framework for HIV AIDS in 2001, which outlines priorities for effectively controlling the epidemic. In accordance with the Framework, the Government launched the “Enhanced HIV*AIDS Control Program” from 2003 to 2008. With the NACP and five provincial programs overseeing the execution of the majority of activities, the Program moved to a decentralized approach [21]. Several other components of the program included improved HIV prevention among the general population through behavior change and communication, safe blood transfusions, and building the capacity of federal, provincial, and non-governmental organizations. CIDA – Canadian International Development Agency funded the capacity-building component for the development of second-generation HIV surveillance and evaluation [21]. A major source of funding for HIV/AIDS programs in Pakistan was the World Bank. Funding the second Social Action Program (1998–2003), it assisted the government in combating HIV/AIDS. Through the HIV/AIDS Prevention Project, the World Bank supported the government's program alongside other development partners (CIDA, DFID, USAID, and UN agencies) [21].

Approximately 54 non-governmental organizations (NGOs) work to raise awareness about HIV/AIDS and provide care and support for those living with the disease [21]. As part of their education and prevention programs, these NGOs also provide education and prevention to sex workers, truck drivers, and other groups at risk [21]. In each of Pakistan's four provinces, they serve as members of the Provincial HIV/AIDS Consortium to coordinate HIV/AIDS prevention and control efforts. Despite NGOs' active involvement in HIV/AIDS prevention, fewer than fifteen percent of the vulnerable are reached [21].

The recent rise in HIV infection could be indicative of a lapse in detection and reporting. A major factor that results in under diagnosis and delayed treatment is the stigma surrounding HIV [22,23]. To tackle this issue, awareness programs centered around basic information on what the virus is, how it affects the body and spreads from individual to individual, and the benefits of prompt treatment and other precautions, need to be held on community level with the help of influential institutions including religious or feudal leaders alongside healthcare professionals. Social organizations can have a significant contribution in the spread of relevant knowledge through social media, door-to-door campaigns and pamphlets to aid prevention, diagnosis and treatment [24].

Setting up a well-connected network of HIV-focused departments or clinics can make testing more robust by making testing facilities available within short radii, as well as incorporating mobile testing facilities for areas without access to such departments. Promotion of self-testing kits can also improve detection rate by allowing people to safely and effectively [25]. Therefore, easy to understand tutorials should be given on a regular basis in high-risk locations. Maintenance of online, digital records and information sharing regarding cases within this network can help isolate areas contributing to transmission and implement localized measures. This is consistent with the 90-90-90 approach issued by The Joint United Nations Programme on HIV/AIDS (UNAIDS) in 2016: 90% individuals knowing their positive status, 90% of aware patients being on anti-retroviral therapy (ART), and 90% patients on ART having achieved viral suppression [26]. While this target for 2020 has not been met, significantly owing to COVID-19 [27], it is a good aim to work towards regarding HIV response.

Following diagnosis, it is essential to ensure provision of appropriate medication and regular follow-up meetings. Immediate intervention not only improves survival and transmission rates but has also shown to improve dropout rates in patients receiving HIV care [28]. With dropout rates as high as 37% on certain accounts, and the consequent risk of ART resistance [29], it is pivotal to develop efficient dispensaries and counseling centers for HIV positive individuals in Pakistan. Moreover, strengthening testing and pre-exposure prophylaxis among adult women in affected areas can help prevent vertical transmission of HIV [30]. According to the Centers for Disease Control and Prevention (CDC), daily oral use of tenofovir/emtricitabine (TDF/FTC) for 1 month before and 1 month after attempted conception is recommended for HIV negative females with HIV positive male partners [31].

Additionally, phylogenetic surveillance can play a vital role in studying genomic variation, risk factors and drug susceptibility during such spikes [32]. Its application proved useful in tracing sources and epidemiology in the HIV-1 outbreak among children in Larkana in 2019, showcasing that upscaling such facilities can improve effectiveness of investigation and planning out interventions in possible epidemics [33]. The financial demand for increasing these facilities in Pakistan can be met in collaboration with WHO or Joint United Nations Programme on HIV/AIDS (UNAIDS).

## 2 Conclusion

The rise in HIV cases, albeit concerning, is unsurprising given the lack of awareness, adequate testing, and patient compliance. With large populations at risk of HIV infection and its complications, notably its progression to AIDS, rapid actions need to be taken to enhance both prevention and treatment before it overwhelms the already struggling healthcare system. The collaboration of healthcare workers and regional administrative bodies can prove useful in engaging communities in order to curtail the disease burden and limit its transmission. Inclusive efforts need to be directed towards at-risk populations including prisoners, children, senior citizens, and pregnant women, also bearing in mind the socioeconomic differences that exist between various settings.

## Ethical approval

N/A.

## Sources of funding

None.

## Author contribution

All authors equally contributed.

## Registration of research studies

1. Name of the registry: N/A
2. Unique Identifying number or registration ID: N/A
3. Hyperlink to your specific registration (must be publicly accessible and will be checked): N/A

## Guarantor

N/A.

## Consent

N/A.

## Declaration of competing interest

None declared.
